# Ninth International Medical Congress, Washington, D. C., September, 1887

**Published:** 1888-04

**Authors:** 


NINTH INTERNATIONAL MEDICAL CONGRESS, WASHINGTON, D. C., SEPTEMBER, 1887.

SECTION XVIII, DENTAL AND ORAL SURGERY.

REPORTED FOR THE INDEPENDENT PRACTITIONER, BY "MRS. M. W. J."

CONTINUED FROM PAGE 138.

### FRIDAY EVENING SESSION.

Dr. A. H. Thompson, of Topeka, Kansas, read a paper entitled "Does Function Control the Evolution of Structure?"

Dr. Thompson referred to a paper on a similar subject read by Dr. C. N. Peirce at the meeting of the American Dental Association at Minneapolis, and brought forward arguments answering his own question in the affirmative. Evolution of function is not isolated from the development of the organ; adaptation to environment is an attribute of function. Organs may be changed, or rudimentary organs developed for the performance of functions before unknown; new organs are developed in response to new wants, or new movements are developed which the wants give rise to; the development of organs is also in ratio to their employment. In the adjustment of means to ends, the tools are adapted to their work. Through this perfect adaptation, the teeth and jaws alone are sufficient to identify a fossil, or to differentiate species. The teeth are so specialized in structure and adaptation. Life depends on the food supply and the ability to utilize it; the teeth are therefore adapted to the food supply, and modified in that adaptation; otherwise great changes in food supply would cause the extinction of species. Gradual changes modify the development, a persistent change in one direction bringing about a corresponding change in the animal structure, adapting it to its environment. Extensive modifications are the result, not of accident, but of compulsory adaptation to food-environment. The use for a thing precedes its growth; the organ is the effect, function the cause; or the preparation of food is the cause, the apparatus the effect. The masticatory apparatus is the most variable, due to the great variability of food; the teeth are developed for a protective purpose, and a special tissue is developed to support them, the whole apparatus becoming more complex according to the nature of food. In the different species, the masticatory area and character changes with the nature of the food. The organs atrophy through disuse, or become mere rudiments, tending to suppression, a subsequent change in habits changing this, and *vice versa.* Hence the inference that physical education will lead to physical improvement, and the importance of teaching habits of mastication, the artificial preparation of our food encouraging idleness in the use of these organs. The suppression of the wisdom teeth and of the laterals is in progress to-day. If the function disappears, can the organ persist?

Dr. W. C. Barrett, who was appointed to open the discussion of this paper, not being present, the discussion was made general.

Dr. W. H. Atkinson being called for, criticised the paper severely as a series of assumptions without reasons behind. What is the purpose of function? Simply the antecedent of request. The mammalian lung was prepared in all its beauty, long before any air enters it. The hydrostatic test of infanticide is infallible; if the lung sinks in water, it has never been inflated; if it has once been inflated, it can never be compressed again. He said the author had gone to the very door of truth, looked through the keyhole, and then sworn there was neither door nor lock, nor keyhole there! In the work of creation all preparation is made beforehand to enable the machine to perform the work lying before it. To study first principles, we must begin with the lowest forms of aggregation—crystallization in the mineral kingdom, cellulation in the vegetable, corpusculation in the animal. The seriated order of consciousness is called mentation; we must seize and comprehend and reduce to milk before we can have nourishment. In the amœba every function is narrowed down to its own individual protoplasmic demand; a mouth is improvised for it, through which pabulum is taken in, enfolded, assimilated, rejected. It feels without nerves, moves without muscles, digests with only a momentary digestive function.

It is a shame for a man to go back on what light he has—saying too deep, too profound. In asking for light, radiance rushes in and fills as far as the real asking goes. If we ask to be filled with only superficial breath, we will get only that; if we ask for thorough inspiration, we will get the cells all filled. Ideas are formed and borne into thought, which become opinion, belief, knowledge, consciousness.

Dr. Thompson—"And yet function does control the evolution of structure!"

A paper was read from Dr. J. B. Davenport, of Paris, France, entitled "Harmony and Discord; Health and Disease; Healers and Hinderers."

He said that the life of the individual was the result of the total functional activity of all the organs of the body, all being vital, though some were more immediately essential than others. He compared the organs of the body to an army, of which the medulla is the general, whose surrender paralyzes the entire force; the heart and lungs are the trusted corps; the nerves are the lines of communication, the glands, muscles, etc., being the common soldiers. In perfect health there is perfect performance of all the functions of all the organs of the entire body; all are in harmonious relation. The slightest failure on the part of any one has its final disastrous result; when two parallel lines begin to diverge ever so slightly, they finally grow very far apart. The failure of one organ weakens others; through their comparative strength, some may exercise vicarious functions for a time, but they finally give way; we learn their weak point only when they break. No new organ will replace those removed by the surgeon. Man needs a varied supply of food. The dentist stands guard over the portals to the digestive laboratory, and should not mar and mangle what is so admirably fitted to do the work.

The beginning of digestion is coincident with mastication and deglutition. The saliva is a true digestive fluid, and we must assume that it is essential to the perfect performance of that operation. Both sides of the mouth should be equally competent to perform their functions. The loss of teeth on one side causes an excessive use of the masticatory muscles on the other. Man has been given thirty-two teeth, because that is the number he needs. The savage uses all his teeth and keeps them in good condition. The teeth of civilized men are more subject to disease than those of the savage; the teeth of Americans are softer than those of English and Scotch. If it was not intended that we should use our teeth, we would have fewer teeth. The size and shape of the jaw is conformable to the natural number of teeth, and so of the glands, blood-vessels, nerves, etc.; they are all related in one general architectural plan. We may think them unimportant, but we should not assume to know the means and ends of creative work. There may be some conditions of life in which the thirty-two teeth are apparently not all needed, but we do not know when they may all be called into requisition. Nature is not so extravagant as to provide against our extractions and excisions, and will not furnish a third dentition to cover the loss of all. When men lose them all it is by the aid of man, or through lack of care. It is not proved that they become less in number or rudimentary, or modified by function.

The importance of the loss of teeth does not lie merely in the loss of masticating surface, but also in the derangement of the remaining teeth, which are robbed of their support, and lose their function through tipping, both occlusion and mastication being ruined. The first molars are needed to support the second and third; the bicuspids, which seem to have but little work to do, serve to preserve the features and to support the other teeth. The race has been much cursed by bad dentistry opposed to natural laws. A man cannot have a series of set rules and adapt each case to these rules. In practice we deal with individuals, not with groups, and must vary according to circumstances and cases. Common sense is the great essential; if our knowledge were as broad as the universe, we might avoid making mistakes.

Dr. W. H. Atkinson having been appointed to open the discussion of this paper said: "In proof-reading we sometimes write on the margin *stet*—I say let it stand."

Dr. Cunningham, of England, said that when Dr. Cravens read his paper on the treatment of pulpless teeth, he had been accorded the privilege of setting before the section some statistics on the subject when his effects arrived. His practice being in a University town, among students who had not the time for repeated dressings and treatment, he was obliged to fill all classes of teeth at one sitting, rarely seeing the patient again. His practice with pulpless teeth was to remove the soft dentine, cleaning out the pulp-chamber, but not the root-canals. When the cavity is ready for filling he places over the entrance to the canals a disk of paper saturated with creosote in which ten or fifteen grains of arsenic has been dissolved. Over this he fills with oxy-chloride and finishes the filling as desired. His success has been so great that he has confidence to assume and recommend this operation. Many of his cases he does not see again, but thinks he would have heard of them if they had been failures, and many others he has under observation that he knows are doing well. This method he calls "immediate root filling." When travelling on the continent recently, he visited the German school at Liepsic, and soon found that they wanted to communicate something which they considered the newest and best. It was "immediate root-filling," so named by Dr. Smith Dodge, but his own cases date back to 1883.

Dr. Cunningham then gave a long list of tabulated cases, statistics of failures and successes under different methods of treament, with different remedies, etc., the conclusion being that the method described in the paper gave by far the largest percentage of successful operations, as far as heard from. He said that he was aware that the pathology of this treatment would occasion debate, but he offered the silent eloquence of facts, and was satisfied with the success. It gives less pain, consumes less time for both patient and operator, and earns the gratitude of the patient. Most of his cases are dismissed in less than half an hour. A broken drill he leaves with impunity, having yet to see the first case where it has caused trouble.

Dr. Kirk, of Indiana, said that seventeen years ago he had been called upon to fill a tooth with a dead pulp. It had to be filled immediately, as the patient was just starting for Kansas. As he never expected to see the man, he decided to do it empirically. He filled with oxy-chloride of zinc, and the patient left, satisfied. In three months he returned, and he dodged him for three weeks, until he came to the office to thank him for the perfect satisfaction the tooth had given him, except for the first three days, when it gave him —— he would not say what. On another occasion he put in, for a bootblack with a swelled face, as an experiment, a gold filling over oxy-chloride of zinc, and the record has been good.

He finds that he has less swelled faces than any one he knows who "treats" after the old methods. He cleans out thoroughly, dries with alcohol and hot air, and fills to the apex with gutta-percha and oxy-chloride of zinc, without treatment. As a rule, a nerve canal that is large enough to be enlarged does not need enlarging; he would keep drills out of canals.

Dr. Ames, of Chicago, said he had been using this method for the past five years. Believes in removal of the contents of canals, thorough disinfecting and immediate filling. He decomposes the contents of the canals by electrolysis, flooding them with an acidulated fluid, using a platinum probe reaching to the apex in the canal. By the action of the current nascent oxygen is liberated, which combines with sulphuretted hydrogen and gives the disinfectant. Then with reamers he removes the contents.

Dr. Conrad, of St. Louis, had acquired confidence in this method by repeated trials and success. He uses peroxide of hydrogen.

Dr. A. E. Baldwin, of Chicago, had not heard Dr. Craven's paper, but he believed in "immediate root filling," even if there is a blind abscess; it can be brought to the surface and treated from the outside. He feared micro-organisms less than Dr. Barrett, and took exception to the statement that there could be no pus without their presence. He believed that more harm than good was done by a multiplicity of medicinal agents. He had been greatly interested in the statements made by the gentleman from England, and had the greatest respect for an open statement of honest opinion.

Dr. Sitherwood, of Bloomington, Ill., was much pleased with the paper read by the gentleman from England. It has been his practice for six years to fill at a single sitting. He believes that success depends on thorough cleansing and germicides.

Dr. Story, of Texas, said that a great variety of treatment had been advocated, but the treatment made but little difference provided the tooth was thoroughly cleaned out and nothing left to decompose. Provided he could get everything *out*, he did not care to get anything *in*. When there has been no pus, he does not care to fill the roots.

Dr. Stack, of Dublin, said he could add nothing to what had been said. In America the people are educated in the proper care of the teeth, and it is comparatively rare to find them so neglected as they usually are when they come under the hands of an English or an Irish dentist. The method advocated in the last paper read seemed a great heresy, but it was supported by a strong array of facts and statistics. The practitioners of America could form no idea of the troubles they, across the water, had to encounter from such a different class of patients.

Dr. Cravens, in closing the discussion of his paper, thanked Dr. Fillebrown for the fair, gentlemanly and scholarly manner in which he had reviewed his paper. One or two points in the discussion demanded reply. He often found teeth that were too tender for him to attempt to touch them the first day, but by giving the patient instructions how to reduce the inflammation and modify the pain, he could then open the pulp cavity and operate with more satisfaction. He was not afraid of the bugaboo of septic conditions corked up. His use of the term "apisal space" had been criticised, but the term was so fixed in dental nomenclature that it was easier to use it than displace it. It was true that he had "failed to mention impenetrable pulp canals," but he did not attempt to mention every possible case. Canals were often too slender to be filled with solids, but if they could be explored they could be filled with shellac on a hog's bristle, using the end out of the skin for the advance end, and leaving it in the canal permanently.

The objection was raised that he filled the roots of deciduous teeth with phosphate of lime, after saying that he used "no medicines." But the cook would indignantly deny having put "medicine" in the biscuits, though she had used salt and yeast powders, both medicinal agents in a certain sense.

The phosphate of lime is a powder, and easily shifted to meet the demands of the organ of absorption. He had chosen his subject after reading the monograph of the Odontological Society of Chicago. He was aware that its reading would be followed by a storm. Men who are eminent as therapeutists or embryologists don't like to see their temple razed to the level of ordinary events. But he asked only a fair trial for his method, with the warning that it requires courage to avow and to follow such a practice.

On motion, a committee of two was appointed to prepare a testimonial to Dr. N. S. Davis, as a mark of appreciation of his services in favor of dentistry.

A vote of thanks was tendered the President of the Section for the able manner in which he had presided over the meeting, overcoming so many obstacles in the most masterly way.

The vote was passed with long and loud applause.

Dr. Taft, in response, said that he appreciated the expression, but that he had fallen so far short of what should have been, that the resolution was unmerited. The credit for the measure of success attained was due, not to himself, but to the members in attendance. The final results were yet to be seen; great fruit must ripen from the seed just sown. Other sections have known but little of what was going on, but the impression is that it has been one of the best of the Congress. It has been unusually well attended. Even at that late hour, after all the other Sections have adjourned, 427 members are present; nearly as many at the last session, at half-past ten at night, as on any preceding occasion. That fact in itself speaks volumes. The effect will not be limited to the dental profession. The deliberations will be published in the transactions, and the physicians who were too busy in their own Sections to know what we were doing will then see the record we have made, and it will be there for us to study whenever we desire. In its social aspects, links have been forged that will not soon be broken. A chord of sympathy has been struck, and pleasant memories will go to our homes with us.

As the President was about to speak the words of adjournment, Dr. Cunningham interrupted him with a resolution of thanks to the Secretaries for their faithful service, and also to the Executive Committee, which had done wonders.

The rooms which had been secured were admirably adapted to the necessities of the occasion. The Franklin School building, in which had been held the clinics, was especially commodious and convenient. There were so many rooms upon the different floors, they were so large and airy, and the light was so ample, that it was difficult to see how better accommodations could have been found. Great credit was due to all the local as well as to the general committees, and Drs. R. F. Hunt, H. B. Noble, M. F. Finley, R. B. Donaldson, Ed. Maynard and D. McFarlan had been untiring in their devotion to the section. Their reward would be in the consciousness of the unqualified approval of all who had been in attendance.

Passed unanimously.

The Section of Oral and Dental Surgery then adjourned, and the Ninth International Medical Congress became a thing of the past.

(A report of the Clinics will be given in the next number.—EDITOR.)

